# Effect of C-Peptide on Diabetic Neuropathy in Patients with Type 1 Diabetes

**DOI:** 10.1155/2008/457912

**Published:** 2008-02-25

**Authors:** Karin Ekberg, Bo-Lennart Johansson

**Affiliations:** Department of Molecular Medicine and Surgery, Karolinska Institute, 17176 Stockholm, Sweden

## Abstract

Recent results indicate that proinsulin C-peptide, contrary to previous views, exerts important physiological effects and shows the characteristics of a bioactive peptide. Studies in type 1 diabetes, involving animal models as well as patients, demonstrate that C-peptide in replacement doses has the ability to improve peripheral nerve function and prevent or reverse the development of nerve structural abnormalities. Peripheral nerve function, as evaluated by determination of sensory nerve conduction velocity and quantitative sensory testing, is improved by C-peptide replacement in diabetes type 1 patients with early stage neuropathy. Similarly, autonomic nerve dysfunction is ameliorated following administration of C peptide for up to 3 months. As evaluated in animal models of type 1 diabetes, the improved nerve function is accompanied by reversal or prevention of nerve structural changes, and the mechanisms of action are related to the ability of C-peptide to correct diabetes-induced reductions in endoneurial blood flow and in Na^+^ K^+^-ATPase activity and modulation of neurotrophic factors. Combining the results demonstrates that C-peptide may be a possible new treatment of neuropathy in type 1 diabetes.

## 1. INTRODUCTION

Neuropathy is one of the most common long-term complications accompanying diabetes mellitus. It affects patients with both type 1 and type 2 diabetes, but it progresses more rapidly and its manifestations are more severe in type 1 diabetes [1, 2]. Diabetic neuropathy is defined by the presence of detectable sensory, motor, and autonomic deficits on clinical examination, with or without the presence of symptoms [3, 4]. As many as 50% of the patients may be asymptomatic, diagnosis may only be made on examination or, in some cases, when the patient presents with a painless foot ulcer [5]. Other patients may not volunteer symptoms but on inquiry admit that their feet feel numb or dead. A thorough neurological examination of the lower limb usually reveals sensory loss of vibration, pressure, pain, and temperature perception mediated by small and large fibers, and absent ankle reflexes. In addition to manifestations of autonomic neuropathy, for example, impaired cardiovascular and gastrointestinal functions, signs of peripheral sympathetic autonomic dysfunction are also frequently seen in patients with diabetes and may include a warm or cold foot, sometimes with distended dorsal foot veins, dry skin, and the presence of calluses under pressure-bearing areas. Diabetic neuropathies may present as rapidly reversible hyperglycemic neuropathy and focal or multifocal neuropathies, but the most relevant clinical form is the persistent distal symmetric polyneuropathy (DSPN) [4].

The DSPN is characterized as a gradual progression in structural changes consisting of distal axonal degeneration of “dying-back” type [6, 7] most prominent in the lower limbs, but involves also small fiber sensory dysfunction early in the course of the condition [7]. The prevalence of DSPN is approximately 30% for diabetes patients in general [8], but the number varies greatly in the literature related to the definition chosen for presence of diabetic neuropathy and the methodology chosen to assess its presence. Clinical examinations and patients'symptom assessment are considered important tools in the evaluation of neuropathy status, but both techniques rely greatly on subjective components and have thus poor reproducibility and specificity. Assessments using more objective markers of polyneuropathy, such as especially nerve conduction velocity (NCV) but also vibration perception threshold (VPT), may serve not only as reliable methods for detection of neuropathy, but the result may also be used for the prediction of mortality in diabetic patients [9, 10]. The pathogenesis of diabetic neuropathy involves metabolic effects mediated directly and indirectly by hyperglycemia, resulting in oxidative stress, accelerated polyol pathway metabolism and generation of advanced glycation endproducts [11–13]. Furthermore, diabetic neuropathy is accompanied by reduced nerve Na^+^,K^+^-ATPase activity, and microvascular abnormalities (e.g., reduced endoneurial perfusion) [14]. Type 1 diabetes is associated with specific structural nerve abnormalities that are not frequent in type 2 diabetes. These abnormalities include axonal atrophy and characteristic nodal and paranodal changes that contribute to the progressive deterioration of nerve conduction velocity [15–17]. In contrast, in type 2 diabetes the axonal degeneration is milder and no or only minimal nodal and paranodal abnormalities occur [17, 18]. However, after several years type 2 diabetes often become insulin and C-peptide deficient, and at this stage it is most likely that the type 2 DSPN will start presenting with characteristics similar to that of type 1 neuropathy.

In the case of type 1 diabetes, available data suggest that C-peptide deficiency is an important contributing factor to the characteristic structural abnormalities [21]. In conformity with this hypothesis several studies have demonstrated that it is possible to retard the progression of diabetic complications by intensified insulin treatment and improved metabolic control, but development of neuropathy cannot be prevented [22–24]. Thus, other factors, such as C-peptide deficiency, are likely to be of importance for the progression of diabetic neuropathy in type 1 diabetes. Evidence documenting significant physiological effects of C-peptide has been presented during the latest decades. It shows that C-peptide, in contrast to previous belief, possesses the characteristics of a bioactive peptide. C-peptide binds specifically to various cell membranes, including endothelial, renal and nerve cells [25], with subsequent activation of an intracellular signaling cascade resulting in stimulation of endothelial nitric oxide synthase (eNOS) and Na^+^,K^+^-ATPase [26]. Moreover, recent data indicate that C-peptide stimulates several transcriptional factors, as well as several neurotrophic factors [27]. Thus, it has been demonstrated that exogenous administration of C-peptide in replacement dose to patients lacking endogenous C-peptide results in restoration of reduced blood flow in several tissues [28–31] and improvement of renal [32] and nerve function, the latter reviewed below. It is also a long-standing clinical observation that compared with type 1 diabetes patients in whom beta-cell secretion ceases totally, those patients who retain a low-endogenous C-peptide and insulin secretion are less prone to develop microvascular long-term complications involving the kidneys, the eyes, or the nervous system [33–35].

## 2. CLINICAL STUDIES ON C-PEPTIDE AND NERVE FUNCTION

To date, only a few studies on the clinical effects of C-peptide on nerve function have been performed, but several preclinical studies indicate significant effects on diabetes-induced nerve dysfunction and structural abnormalities [36–38]. The available clinical results indicate beneficial effects of C-peptide on both peripheral and autonomic nerve function in type 1 diabetes patients. Thus, in a double-blind, placebo-controlled study including 46 type 1 diabetes patients, with an average age of 29 years and approximately 10 years of diabetes duration, and with reduced sensory nerve and motor nerve conduction velocities (NCV) but no other signs of neuropathy, C-peptide replacement (1.8 mg/day) or placebo was given for 3 months together with the patients' regular insulin therapy [19]. Sensory nerve conduction velocity assessed in the sural nerve bilaterally, but not motor NCV (peroneal nerve), increased gradually during the study. The increase after 3 months amounted to 2.7 m/s, corresponding to an 80% correction of the initial conduction velocity deficit in these patients. This change was accompanied by an improvement in vibration perception assessed on the dorsum of the feet, although these patients had essentially normal perception thresholds already at baseline. This improvement is consistent with an improved sural nerve function since vibration perception in this anatomic region is primarily mediated via the large fibers of the sural nerve.

**Figure 1:**
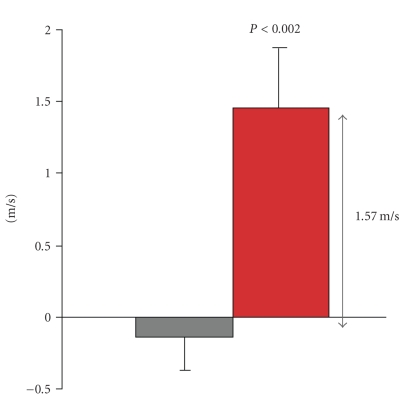
Change in peak sensory nerve conduction velocity in the sural nerves following 3–6 months of C-peptide replacement treatment (red bar) or placebo (gray bar) to patients with type 1 diabetes. The figure presents pooled data from [19, 20].

The improvement in nerve function demonstrated in this early patient population is now confirmed and extended in a recently completed clinical trial including patients with diabetic neuropathy [20]. The study was a double-blind, placebo-controlled, randomized multicenter study, including 161 type 1 diabetes patients, with an average age of 44 years and 29 years of diabetes duration and defined DSPN (according to the San Antonio criteria [3]). At baseline their sensory nerve conduction velocity assessed in the sural nerve (SCV) was on average 2.6 SD below normal, and following 6 months of C-peptide replacement treatment there was a statistically significant improvement in SCV for the patients receiving C-peptide, amounting to 0.48 ± 0.19 m/s assessed as peak velocities and 0.93 ± 0.29 m/s for the initial response (the velocity change from baseline for the fastest axon in the nerve). However, these changes were not statistically significantly different from the change in the placebo group. The number of responders, defined as patients with an improvement in peak SCV > 1 m/s, was statistically significantly greater in the group receiving C-peptide as compared to those receiving placebo (37% versus 19%, resp., *p* < .032). It is noteworthy though that among the included patients some had substantial nerve conduction deficits at baseline and with the study duration of no more than 6 months it is conceivable that the patients who were relative less affected at baseline may have a greater potential for improvement. Thus, a subgroup analysis was performed in the subset of patients with the least affected SCV at baseline (half of the patient population). In this group, C-peptide administration for 6 months resulted in an improvement that was 1.03 m/s greater as compared to the change in the corresponding placebo treated patients (*P* < .014). Analyzing the number of responders in this half of the patient population revealed that among patients receiving C-peptide, 39% demonstrated an improvement in SCV >1 m/s whereas only 5% among the placebo treated patients showed a similar change *P* < .004. Accompanying these changes in sural nerve conduction velocity there where, however, no statistically significant change in motor nerve conduction velocity, but there was an improvement within the C-peptide treated patients for vibration perception. There was also a trend towards an improvement in neurological examination scores following C-peptide. Combining these data provides evidence of a therapeutic improvement of diabetes-induced peripheral nerve dysfunction following C-peptide administration in patients with type 1 diabetes; 3–6 months of C-peptide replacement to patients with early stage neuropathy resulted in approximately 1.5 m/s in sensory nerve conduction velocity (see Figure 1) accompanied by other signs of nerve function improvements.

There is also evidence of beneficial effects of C-peptide administration to type 1 patients with signs of autonomic neuropathy. Deficient autonomic nerve function may be evaluated in patients as reduced heart rate variability (HRV) during deep breathing, a measurement that, with a high degree of reproducibility, primarily reflects vagal function. Patients were studied twice under normoglycemic conditions and during 3-hours intravenous infusion of either human C-peptide or saline in a double-blind study. At baseline HRV was reduced 13 ± 1% (normal reference value 24%) and during the C-peptide infusion which restored plasma concentrations to physiological levels, the HRV improved to 20 ± 2%, while no change was seen after saline infusion (*p* < .001) [39]. The heart rate brake index after a tilting maneuver was also improved after C-peptide for 3 hours in the patients showing reduced index before the study. In agreement with these results, a 20% improvement in HRV was seen after 3 months of C-peptide replacement in type 1 diabetes patients whereas no change or a slight deterioration was observed in the same patients during a placebo treatment period [32].

## 3. TREATMENT OF DIABETIC NEUROPATHY

There is no effective pharmaceutical therapy available for diabetic neuropathy today. The onset and the progression of the diabetes-induced abnormalities may be delayed by maintenance of good glycemic control [22–24]. In the DCCT, the incidences of diabetes neuropathy were substantially lower in patients on intensive insulin treatment as compared to conventional insulin therapy [23]. For the patients in the DCCT secondary prevention group, with an average age of 28 years and a diabetes duration of 9 years, the 2% improvement in HbA_1c_ seen following 5 years of intensified insulin treatment was accompanied by an improved SCV of 1.5 m/s whereas patients with unchanged metabolic control in the conventional treatment group experienced a 2.2 m/s reduction in SCV. Interestingly, the patients in this group are directly comparable to the patients in the first C-peptide intervention study (average age 29 years and diabetes duration of 10 years) where 3 months of C-peptide replacement resulted in 2.7 m/s improvement in SCV, and it is of note that these patients were already on an intensified insulin treatment regimen [19]. The magnitude of the response following C-peptide replacement treatment occurred completely independent of improved glycemic control, and in fact in top of an already good glycemic control. This suggests that the C-peptide is in fact acting on DSPN disease-modifying mechanisms. Impaired nerve blood flow secondary to perturbed nitric oxide metabolism [36, 38] and reduced levels of nerve Na^+^,K^+^-ATPase activity [40, 41] are both factors that have been implicated in the pathogenesis of the DSPN [7]. The ability of C-peptide to improve endoneurial blood flow and Na^+^,K^+^-ATPase activity as well as its stimulation of neurotrophic factors as demonstrated in several animal models of type 1 diabetes [27, 37, 40] is thus likely to contribute to the positive effects of the peptide.

In addition to improved insulin therapy, most therapeutic interventions previously evaluated for DSPN have been directed toward correction of the adverse effects of hyperglycemia. For example, one approach involves the reduction of the intracellular sorbitol accumulation by aldose reductase inhibitors (ARI). Although shown to have beneficial effects on neuropathy [42, 43], several clinical trials involving ARIs have been discontinued because of unacceptable adverse effects, for example, skin rash, renal toxicity, and serious hepatic effects [44]. A new and apparently well-tolerated ARI, ranirestat, is currently in development, and phase II data indicate that its administration for 60 weeks to mostly type 2 diabetes patients has beneficial effects on nerve function [45, 46]. Another compound also aiming to minimize the effects of hyperglycemia is the specific protein kinase C beta inhibitor, ruboxistaurin, but recent clinical development has not documented impressive clinical effect [47].

It has become increasingly apparent that DSPN presents a clear unmet medical need, and the health authorities and representatives for patient associations have expressed their concern [5]. Previously, the regulatory agencies have required efficacy on symptom relief and reduced wound and amputation frequency in order to accept a new drug application. However, it is now becoming increasingly recognized that treatment should be started well, before the deterioration of nerve function has reached the stage of severe symptoms and wounds. Moreover, the treatment should not only be directed at a symptom relief but to modify the underlying disease mechanisms. In the case of peripheral neuropathy accompanying type 1 diabetes, the beneficial effects on nerve function following C-peptide replacement therapy may indicate a new potential treatment paradigm, even though extended clinical trials will be needed to finally elucidate its usefulness.
